# Genomics of psychiatric disorders: Regional challenges and opportunities

**DOI:** 10.7705/biomedica.6996

**Published:** 2023-03-30

**Authors:** Diego A. Forero

**Affiliations:** 1 Facultad de Ciencias de la Salud y del Deporte, Fundación Universitaria del Área Andina, Bogotá, D.C., Colombia dforero41@areandina.edu.co Fundación Universitaria del Área Andina Fundación Universitaria del Área Andina Bogotá, D.C. Colombia dforero41@areandina.edu.co

Research in the etiology of psychiatric disorders is a major global area in the health sciences, considering their prevalence and the large need for knowing more about their biological basis ^1^. In this editorial, I will briefly discuss major advances in genomics of psychiatric disorders and will highlight key regional challenges and opportunities.

A recent article, based on the Global Burden of Disease Study 2019, estimated that about 970 million people were affected around the world by common psychiatric disorders and that anxiety and depressive disorders led to the largest numbers of disability-adjusted life-years (DALY) ^2^. A previous work analyzed the prevalence of psychiatric disorders in fourteen countries and found that México and Colombia had higher rates than several European countries ^3^. In terms of the global economic impact associated with psychiatric disorders, a recent paper estimated that it is around USD $5 trillion; for the global burden of disease region in which Colombia is located it is equivalent to 5.7 percent of the gross domestic product (GDP) ^4^. These major impacts on burden of disease, particularly on morbidity, is associated with the fact that common psychiatric disorders affect patients for many decades of life ^2^.

## Global advances in genomics of psychiatric disorders

It has been known that psychiatric disorders have a genetic basis ^1^. Examples of estimated heritability for major psychiatric disorders are: 0.79 for attention-deficit hyperactivity disorder, 0.77 for schizophrenia and 0.68 for bipolar disorder ^1^. Major advances in psychiatric genomics research come from two main areas: 1) Development and implementation of novel methods for genomic analysis, such as microarrays and next-generation sequencing ^5^, and 2) International and interinstitutional collaborations allowing the analysis of tens of thousands of patients and control subjects ^6^. Examples of commonly used designs in psychiatric genomics are the genome-wide association studies (GWAS) ^7^, the genome-wide expression studies (GWES) and the epigenome-wide association studies (EWAS) ^5^.

As an example of a recent international advance in psychiatric genomics, a meta-analysis of GWAS for depression included data from more than 340,000 cases and from more than 814,000 control subjects of European ancestry, which were previously genotyped for hundreds of thousands of single nucleotide polymorphisms (SNP's) ^8^. They identified 178 genomewide significant loci, providing novel top genes, such as the neuronal growth regulator 1 (*NEGR*1) ^8^. Another recent international example of interest reported the meta-analysis of GWAS for neuroticism ^9^, a personality trait associated with depression and anxiety, including data from more than 449,000 subjects of European descent. They identified novel significant associations for more than 500 genes ^9^, highlighting in addition the large potential of the genomic analysis of endophenotypes related to psychiatric disorders.

In the context of global initiatives, the Psychiatric Genomics Consortium (PGC) (pgc.unc.edu) has promoted international collaborations (from an open science perspective), between scientists from more than 40 countries, in major psychiatric disorders, allowing the joint analysis of tens of thousands of patients and control subjects ^6^. Two major international examples of large research initiatives, which have led to dozens of international publications, are the UK Biobank and the All of Us Research Program.

The UK Biobank (ukbiobank.ac.uk) has evaluated around 500,000 participants from the United Kingdom, including information from health electronic records, among other phenotypic data, and hundreds of thousands of SNP, which can be accessed for secondary analysis ^10^.

The All of Us Research Program (allofus.nih.gov) plans to have about one million participants from the United States of America (with more than 300,000 subjects recruited), with detailed phenotypic and genotypic data available ^11^. In recent years, it has been highlighted that there is a major preponderance of individuals of European and North American countries as participants in genomic studies, with a very low participation (less than one percent of the total) of subjects from Latin American countries ^12^.

## Regional challenges and opportunities

There are several opportunities for the strengthening of research in genomics of neuropsychiatric disorders in Latin America ^13^. There is the need for the consolidation of international and interinstitutional consortia (promoting public/private partnerships) in the region, to facilitate the sharing of resources and to achieve the large sample sizes currently needed in the field, from the perspective of open science. In this context, the Latin American Genomics Consortium (LAGC) ^12^ has been recently created as a major and inclusive initiative in the region. As genomics involves expensive high-throughput platforms, adequate local funding for those analysis is key, highlighting the importance of having research in mental health as a priority from the governments ^13^. Longitudinal studies, such as cohorts, will benefit from increased funding. In the context of the need of novel treatment strategies, it is well known that biomedical sciences are fundamental for the drug discovery processes, which require years and large budgets ^14^.

Advanced training in high-throughput data analysis is a global need in genomics and it should be included in the curricula of regional master of sciences and doctorate programs in the biomedical sciences ^15^. In addition, an adequate teaching about the molecular basis of human diseases, in the context of the current high relevance of precision medicine ^16^, should be considered as a key element in the curricula of updated undergraduate programs in the health sciences ^17^. Strategies aimed at strengthening research training of mental health professionals (psychiatrists and psychologists) and at increasing protected time for scientific activities in clinical institutions would be helpful to provide the needed regional structure of clinical research in mental health ^13^. As the translation of genomic findings to the identification of potential targets of novel pharmacological treatments depends on functional studies ^18^, there is the potential for the regional development of further studies in animal and cellular models.

Future research in the genomics of psychiatric disorders in our region will contribute to a further understanding of the molecular basis of entities of major local impact in mental health ^13^, in addition to providing additional diversity, in terms of patients from other ancestries ^12^, to the international efforts in psychiatric genomics, giving more visibility to the strategies from the Global South.
